# Green industrial policy and green development of agriculture—Quasi-natural experiment based on the Yangtze River Economic Belt in China

**DOI:** 10.1371/journal.pone.0308307

**Published:** 2024-09-18

**Authors:** Jingbo Shao

**Affiliations:** 1 Economic Institute, Guizhou University of Finance and Economics, Guiyang, China; 2 International Tourism Culture College, Guizhou Normal University, Guiyang, China; Ural Federal University named after the first President of Russia B N Yeltsin Institute of Physics and Technology: Ural’skij federal’nyj universitet imeni pervogo Prezidenta Rossii B N El’cina Fiziko-tehnologiceskij institut, RUSSIAN FEDERATION

## Abstract

Based on the panel data of 30 provinces, municipalities, and autonomous regions in China from 2012 to 2022, from the perspective of harmony between man and nature, this paper selects 20 indicators to measure the level of agricultural green development from five dimensions such as ecological conservation and resource conservation by entropy weight method. On this basis, taking the implementation of green industrial policy in the Yangtze River Economic Belt as a quasi-natural experiment, the policy effect of green industrial policy on agricultural green development was analyzed by using the difference-in-difference method. The study found that: (1) the green development of agriculture is basically increasing year by year in each province, but there are some differences in the green development of agriculture among provinces; (2) Compared with the non-implementation areas of policies, the green development of agriculture in the implementation areas of policies has been significantly improved, and has passed a series of robustness tests; (3) The mechanism analysis shows that the green industrial policy has a crowding-out effect on industrial development, but significantly enhances the ecological construction and protection, thus promoting the green development of agriculture; (4) Heterogeneity analysis shows that the policy has a significant positive incentive effect on the upper and lower reaches of the Yangtze River Economic Belt, and the incentive effect on the middle reaches is not significant; (5) The impact of technological level on agricultural green development shows a positive U-shaped relationship. The improvement of education and information development levels also effectively promotes the green development of agriculture. This paper provides important empirical evidence and factual references for further promoting the green development of agriculture and the improvement of green industrial policies.

## Introduction

As a basic strategic industry, agricultural development is related to farmers’ livelihood, rural prosperity and national stability [1]. Since the reform and opening up, China’s agriculture has achieved remarkable development achievements, and the agricultural production capacity and industrialization level have been steadily improved [2]. However, China’s agricultural development mainly depends on excessive resource consumption for a long time, and the rural ecological environment has been red light [3, 4]. In particular, the extensive use of chemical fertilizers and pesticides has brought a serious burden to the ecological environment [5]. The supply of high-quality agricultural products cannot meet the people’s growing needs for a better life [6–9]. How to transform the mode of agricultural development, optimize the agricultural structure, and transform the kinetic energy of agricultural growth? The comprehensive green transformation of agricultural development answers this question, and provides a direction for China to promote agricultural development [10], improve agricultural comprehensive benefits, enhance the ability of sustainable agricultural development, and realize the transformation from a large agricultural country to a strong agricultural country..The green transformation of agriculture is not only the inherent requirement of high-quality agricultural development but also the key link to promote the green transformation of the whole economy and society [11]. In this regard, the Chinese government has also introduced a series of policies. in 2012, the 18th National Congress of the Communist Party of China proposed to increase the construction of ecological civilization based on the original “four in one” and focus on promoting the overall layout of the “five in one” development of green development. How to promote the green development of agriculture has aroused thinking in the academic circle. In 2022, the 20th National Congress of the Communist Party of China proposed that adhering to the harmonious coexistence of man and nature is the mission of the Communist Party of China. Promoting the green and low-carbon development of economic and social development is the key link to achieving high-quality development. In the same year, Comrade Xi Jinping pointed out that the agricultural power is the foundation of the socialist modernization power in the Central Rural Work Conference, highlighting the importance of high-quality agricultural development [1, 12]. The introduction of relevant policy measures has created a good institutional environment for accelerating the overall green transformation of agriculture. As a top-level design, the agricultural policy profoundly reflects the government’s choice of agricultural development direction and development focus. Analyzing the focus of AGD policy is effective feedback on the implementation of existing policies [13], which affects the efficiency output of agricultural policy to a certain extent. Therefore, in the process of agriculture seeking green development, it is worth exploring the influence mechanism of green industrial policy on AGD from the perspective of green industrial policy implementation.

In September 2018, the Ministry of Agriculture and Rural Affairs of the People’s Republic of China issued the “Implementation Opinions of the Ministry of Agriculture and Rural Affairs on Supporting the Green Development of Agriculture and Rural Areas in the Yangtze River Economic Belt” to promote the AGD and rural areas in the Yangtze River Economic Belt and put forward detailed development opinions. To explore the policy effect and influence mechanism, this paper takes the implementation of the “Implementation Opinions of the Ministry of Agriculture and Rural Affairs on Supporting the Green Development of Agriculture and Rural Areas in the Yangtze River Economic Belt” as a quasi-natural experiment. Based on the relevant data of 30 provinces, municipalities, and autonomous regions in China, the difference-in-difference method (DID) is used to compare the impact of policy implementation on AGD by comparing the policy implementation areas and non-implementation areas before and after policy implementation. Firstly, clarify the theoretical mechanism of green industrial policy affecting agricultural green development, and on this basis, put forward research hypotheses. Secondly, the index system of AGD is constructed and quantified. Thirdly, by constructing an econometric model, this paper empirically tests the influence mechanism of green industrial policy on AGD, analyzes the influence of mechanism variables, and further explores the heterogeneity of its influence. Finally, according to the research conclusions, the countermeasures and suggestions to promote the AGD are put forward.

The remaining part of this article is organized as follows. Section 2 Literature review and research hypothesis, while Section 3 is research methods, variables, and data, focusing on the construction of the index system, in Section 4 the empirical results are analyzed, and a robustness test is conducted, Section 5 is mechanism analysis, Section 6 discusses heterogeneity to explore the differences in policy effects between regions, and finally, Section 7 summarizes the research and puts forward countermeasures and suggestions.

## Literature review and theoretical lenses

### Literature review

The existing research results on AGD are relatively rich, which lays a solid foundation for this study. It mainly reflects the concept interpretation, index construction, attention to the region and related influencing factors, and related policy analysis of AGD.

#### Concept interpretation and index construction

The Green Revolution is often seen as the epitome of the dawn of technological progress and modernization in the agricultural sector of developing countries [14, 15]. In a broad sense, green development is an ecological development under the concept and strategy of “Continual Development” [16, 17]. On this basis, scholars have systematically studied the connotation of AGD. AGD usually takes agricultural green products as the core, green technology as the driving force, and green policy as the guarantee, and promotes the coordinated development of agricultural ecological, social, and economic benefits [18]. Some scholars believe that the AGD is to achieve green development in all aspects of the agricultural production environment, production process, and the quality of agricultural products [15, 19]. Other scholars believe that we should follow the law of ecological development, make rational use of ecological resources, realize the self-recycling of resources, and emphasize the importance of the ecological environment to the AGD [20]. In general, scholars believe that AGD is a development mode that focuses on agricultural resource conservation and rural environmental protection [21], and provides high-quality agricultural products to meet the growing needs of the people for a better life. Based on clarifying the connotation of AGD, scholars have focused on the evaluation of the AGD level. For the evaluation index system, The existing research mainly quantifies the dimensions of resource conservation, environmental friendliness, ecological conservation, economic growth, and food security [22, 23], analyzing the inter-provincial and annual AGD and the differences between various dimensions [24]. For evaluation methods, the entropy weight method [10], analytic hierarchy process [25], and linear weighting method [2] are often used to evaluate the level of AGD.

#### Attention area and related influencing factors

Clarifying the spatial and temporal characteristics and driving factors of agricultural green GDP will help the spatial economic theory to explain the spatial agglomeration of economic factors [26]. For the concerned areas, the existing scholars mainly focus on the AGD in provinces [1], the Yangtze River Basin [27], and the Yellow River Basin [28]. Some scholars have explored the impact mechanism of AGD in different regions, and believe that the inter-provincial development of AGD is significantly different, and there are obvious spatial spillover effects [1, 2, 11, 29, 30], but the analysis methods or impact mechanisms are different. For example, Deng et al. (2022) mainly used the spatial Dubin model to explore the spatial and temporal evolution characteristics and spatial spillover effects of agricultural green technology in China [29]. Chen et al. (2021) mainly used the spatial correlation network structure to explore its inter-provincial spillover effects [11], and so on. However, many factors affect the AGD. For example, agricultural insurance is a common means to promote the AGD. It can not only encourage farmers to adopt green production technology and improve production efficiency but also achieve the purpose of reducing chemical input and protecting the environment [31]. The shape effectiveness of the environmental Kuznets curve is also different between developed and developing groups [32, 33]. From a policy perspective, it is recommended that governments formulate relevant policies to prioritize the efficiency of raw material resources in the agricultural sector [34] and promote green agricultural development. The impact of the integration of agricultural ecological efficiency can not be ignored [35, 36]. It has a significant effect on promoting the sustainable development of agriculture [37] and further promotes the adjustment, optimization, transformation, and upgrading of agricultural industrial structures [38]. New agricultural cooperation [27], agricultural green technology innovation [39], regional exchange of green technology [19, 40], appropriate subsidy policies [41], and digital economy [42–44] all have effectively promoted the AGD [45, 46].

#### Policy analysis

The empirical research on the policy analysis of AGD mainly includes quasi-natural experiments based on high-standard farmland construction policies [47] and quasi-natural experiments in the “two-oriented society” pilot area [48]. Under the challenge of various ecological security incidents, it has become the consensus of most countries that agriculture should be transformed in the direction of green ecology. The EU’s common agricultural policy is one of the earliest agricultural policies in the world to promote the AGD [49]. The important purpose of the introduction of AGD support policies is to enhance the ability of sustainable agricultural development and the synergy between ecology and development. It advocates increasing the number of green support policy tools, and it is important to establish an effective implementation, supervision, and feedback mechanism [50].

In general, the relevant achievements on AGD are relatively rich. Scholars’ research on AGD mainly focuses on the spatial effect of green development, the measurement of AGD, and the analysis of influencing factors. The existing literature lacks research on the impact mechanism of green industrial policy on AGD. In summary, compared with the existing research, the marginal contribution of the research: (1) Based on the new perspective of harmonious coexistence between man and nature, this paper creatively constructs a five-dimensional index system of AGD level with ecological conservation, green supply, economic growth, resource conservation, and environmental friendliness. (2) The difference-in-differences method is used to analyze the policy effect of green industrial policy on AGD. This paper enriches the relevant literature on the impact of policy on AGD. (3) In-depth investigation of the heterogeneous impact of policies on different regions of the Yangtze River Economic Belt.

### Theoretical lenses and research hypothesis

The internationally accepted theory of sustainable development refers to the development that not only meets the needs of contemporary people but also does not harm the interests of future generations [51]. Sustainable development is divided into weak sustainable development and strong sustainable development [52, 53]. In 2015, the concept of green development, one of China’s “five new development concepts” (innovation, coordination, green, openness, and sharing), was formally proposed at the Fifth Plenary Session of the 18th CPC Central Committee. Green development and sustainable development are ideologically the same. It is not only the inheritance of sustainable development, but also the theoretical innovation of sustainable development in China [54]. The report of the 20th National Congress of the Communist Party of China mentioned that Chinese modernization is the modernization of harmonious coexistence between man and nature. How to solve the harmonious coexistence between man and nature is the main problem facing green development. The AGD is an important topic in the study of industrial greening. Under the guidance of the government’s agricultural green policy, to a certain extent, it can improve people’s awareness of green development [55], change the concept of extensive agricultural development, and promote green awareness spillover [56]. The concept of green development further guides the demand for agricultural green consumption, thereby stimulating agricultural green production behavior [57]. Therefore, this paper puts forward the hypothesis:

Hypothesis 1: Green industrial policy can improve the level of AGD.

The green industrial policy requires industrial development to take the road of clean production [41], and industrial development is usually accompanied by high pollution and high consumption [58]. Therefore, it is speculated that green industrial policy has a certain negative effect on industrial development. Through the crowding-out effect on industries, especially high-polluting industries, the development of green agriculture can receive more policy tilt and attention [59, 60]. In addition, since the 18th National Congress of the Communist Party of China, the Party Central Committee has placed the construction of ecological civilization in an important position in the overall work, and the green industrial policy aims to guide and urge the industry to green, low-carbon and ecological development [41, 61]. The transformation of green development from concept to practice requires the support of production factors such as relevant capital, and the improvement of ecological construction and protection can lay the foundation for green development [62]. In summary, the green industrial policy promotes the AGD by weakening industrial development and improving ecological construction and protection. Therefore, this paper proposes hypothesis 2 and hypothesis 3.

Hypothesis 2: Green industrial policy has a crowding-out effect on industrial development.
Hypothesis 3: Green industrial policy can improve ecological construction and protection.

**Fig 1:**
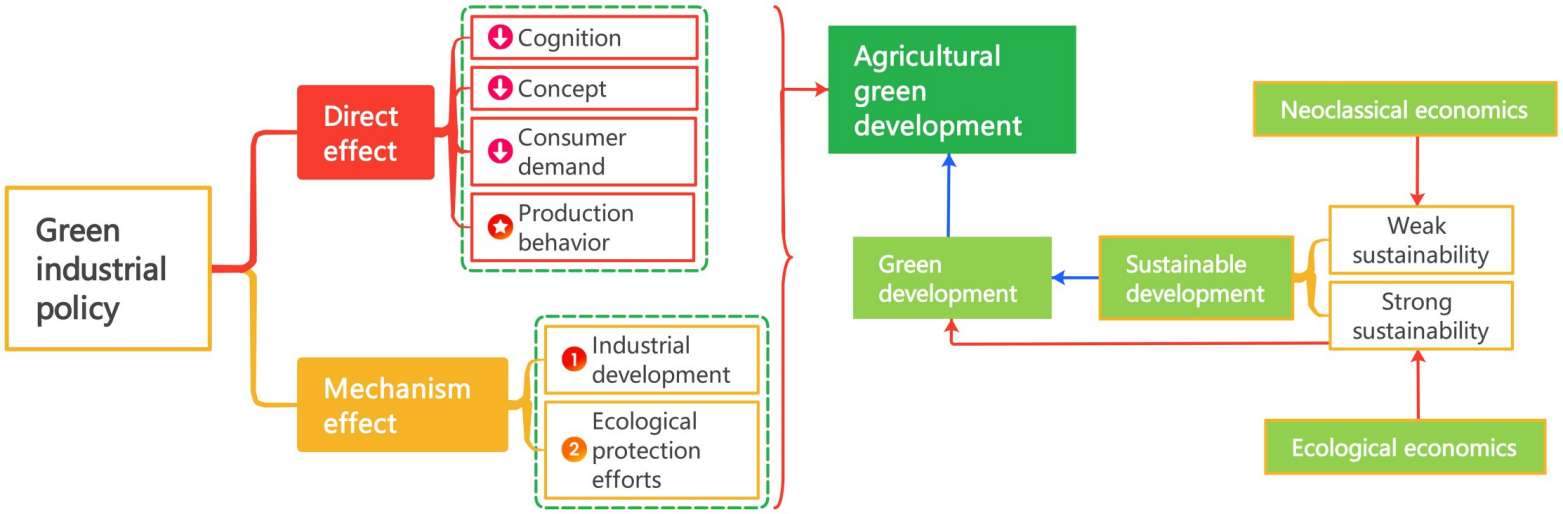
Theoretical context diagram and mechanism. It combs the theoretical context and theoretical mechanism of this paper. This figure is generated in MindMaster.

The theoretical context is shown in Fig 1.

## Research methods, variables, and data

### Research methods

#### Entropy weight method

This paper follows the principles of representativeness, comparability, dynamics, and operability of index selection, screens AGD indicators, and establishes an index system. Due to the differences in the dimension, order of magnitude, and positive and negative orientation of each index, it is necessary to standardize the initial data. In the multi-index comprehensive evaluation, the determination of the weight of the evaluation index is related to the accuracy and credibility of the empirical results. To avoid the deviation of subjective weight determination methods such as the expert consultation method, this paper uses the objective entropy weight method to calculate the index weight and calculate the comprehensive score of AGD based on the weight coefficient. Since the entropy weight method has been relatively mature, the specific details refer to the study of Tan and Qi (2023) [20].

#### Difference-in-difference method

The DID refers to the calculation of the incremental difference between the “experimental group” and the “control group” [63] in the natural experiment under the intervention by using the data of observational learning. The DID method [64] can be understood as a simulation of random assignment experiments to verify causality without random experiments. It is divided into the following steps:

Firstly, for a natural experiment, divide all the sample data into two groups: one group is affected by the intervention, that is, the experimental group; the other group is not affected by the same intervention, that is, the control group.

Secondly, select a target index to be observed. The target index of this paper is AGD.

Thirdly, the difference between the two groups was obtained by twice difference (subtraction) before and after the intervention, representing the relative relationship between the experimental group and the control group before and after the intervention.

Fourthly, the second difference between the two groups was performed to eliminate the original difference between the experimental group and the control group, and finally, the net effect of the intervention was obtained.

This paper takes 11 provinces in the Yangtze River Economic Belt as the treatment group and the remaining 19 provinces as the control group to examine the impact of green industrial policies on the AGD in the Yangtze River Economic Belt. From the time dimension and the regional dimension, the difference-in-difference method is used to compare the development differences between the regions before and after the implementation of the policy and between the regions where the policy is implemented and the regions where the policy is not implemented and to exclude the influence of factors that do not change with time as much as possible. Therefore, the following DID model [63] is constructed as shown Eq (1).


$${AGD}_{it}=\alpha +\beta {DID}_{it}+\rho X_{it}+\mu_{i}+\lambda_{t}+\varepsilon_{it}$$ (1)

Where: *i* denotes province, *t* denotes year. *AGD*_*it*_ is the explained variable; *DID*_*it*_ represents the policy dummy variable, which is obtained by the interaction between the dummy variable *treat*_*i*_ and the time dummy variable *time*_*t*_. If it belongs to the Yangtze River Economic Belt, *treat*_*i*_ is recorded as 1, otherwise, it is recorded as 0. Similarly, the *time*_*t*_ value before the policy shock (before 2018) is 0, and the *time*_*t*_ value after the policy shock is 1; *X*_*it*_ represents a series of control variables. In addition, the article further controls the time and individual fixed effects; *ε*_*it*_ represents the random error term.

### Variable declaration

#### Explained variable

Combined with the theoretical context of AGD, the index system of dependent variables is constructed from the five dimensions of resource conservation, environmental friendliness, ecological conservation, green supply, and economic growth. Firstly, by constructing a resource-saving indicator system, we can quantitatively evaluate the efficiency of resource utilization in the process of agricultural production, and promote the development of agriculture in a more efficient and resource-saving direction [13]. Secondly, the construction of an environment-friendly index system can evaluate the impact of agricultural production activities on the environment, guide agricultural producers to adopt more environmentally friendly production methods, and reduce the negative impact of agricultural production on the environment [65]. Thirdly, the construction of ecological conservation index system can evaluate the impact of agricultural production on the ecosystem and promote the harmonious coexistence of agriculture and ecosystem [66]. At the same time, ecological conservation is also an important way to improve the quality of agricultural products and the comprehensive benefits of agriculture. Fourthly, the construction of a green supply index system can evaluate the quality and safety of agricultural products [67], promote agricultural producers to improve the quality and added value of agricultural products, and meet consumers’ demand for green, healthy and high-quality agricultural products. Fifthly, the AGD should not only pay attention to ecological environmental protection and resource conservation, but also achieve economic growth and increase farmers’ income [68]. The construction of economic growth index system can evaluate the economic and social benefits of AGD, provide decision-making basis for policy makers, and promote the virtuous cycle of AGD and economic growth.

Among them, resource conservation, environmental friendliness, and ecological conservation represent AGD at the level of natural ecological interests, while green supply and economic growth represent that at the level of human interests. The harmonious coexistence between man and nature requires a resource-saving and environment-friendly development path [69], which is the essential characteristic of the human and natural life and development community, and the inherent attribute of AGD. Ecological conservation is helpful in improving the regional ecological environment and promoting the harmonious coexistence between man and nature [70], which is the fundamental requirement of AGD. Under the background of food security, green supply explores the level of agricultural products from the aspects of green output and food supply of agricultural products, which is the fundamental purpose of AGD [71]. Economic growth and environmental protection cannot be ignored, which is an important goal of AGD [72]. Therefore, from the perspective of economics as well as the harmonious coexistence between man and nature, the level of AGD (represented by a “score”) is measured and shown in Table 1.

**Table 1:** Index system of AGD level.

Dimension	Secondary Index	Unit	Measure	Attribute	Weights
Economic growth	The first industry-added value of annual growth rate	%	The first industry-added value / GDP	+	0.009
	Total agricultural output per unit of sown area	100 million yuan / thousand hectares	Gross agricultural output value / sown area	+	0.001
	Labor productivity	100 million yuan / ten thousand people	Total agricultural output / total agricultural population	+	0.023
	Proportion of agricultural financial support	%	Agricultural fiscal expenditure / total expenditure	+	0.122
Ecological conservation	Forest coverage	%	Forest coverage rate	+	0.001
	Soil erosion control acreage	Thousand hectares	Soil erosion control area	+	0.018
	Agricultural disaster resistance index	%	(Crop area-crop affected area) / Crop area	+	0.001
	Control rate of crop diseases, pests, weeds, and rodents	%	Crop pest control area / crop pest occurrence area	+	0.003
Resource-saving	Total power of agricultural machinery per unit of sown area	Ten thousand kilowatts / thousand hectares	Total power of agricultural machinery / total sown area	-	0.073
	Multiple Cropping Index of Cultivated Land		Sowing area / cultivated area	-	0.109
	Proportion of water-saving irrigation area	%	Water-saving irrigation area / total irrigation area	+	0.012
	Per unit water consumption of agricultural output value	Billion cubic meters / billion yuan	Agricultural water consumption / the total agricultural output value	-	0.094
Environmentally friendly	Intensity of agricultural fertilizer application	Thousand tons / thousand hectares	Agricultural fertilizer amount / sown area	-	0.061
	Use intensity of agricultural diesel oil	Thousand tons / thousand hectares	Agricultural diesel quantity / sown area	-	0.095
	Pesticide use intensity	Thousand tons / thousand hectares	Pesticide use / sown area negative	-	0.134
	Usage strength of agricultural plastic film	Thousand tons / thousand hectares	Agricultural film dosage / planting area	-	0.042
Green supply	Level of green food certifications	Piece	Number of green food certifications	+	0.146
	Level of Organic food certification	Piece	Organic food certification quantity	+	0.033
	Level of geographical indication certification of agricultural products	Piece	Number of geographical indication certification of agricultural products	+	0.017
	Grain product growth rate	%	(Grain yield in this year-that in the previous year) / grain yield in the previous year	+	0.006

#### Core explanatory variable and control variables

The core explanatory variable is DID, which is obtained by the interaction between the virtual variable *treat*_*i*_ and the time virtual variable *time*_*t*_. In addition, this paper selects education level (‘edu’), information development level (‘inf’), and technical level (‘tec’) to represent social influencing factors. The level of education is expressed by the proportion of graduates from ordinary colleges and universities in each region accounting for national totality. The level of information development is expressed by the proportion of the amount of regional post and telecommunication businesses accounting for the national totality. To investigate the nonlinear relationship of technical levels, this paper introduces the square term of technical level (‘tecc’). The technical level is expressed by the proportion of the number of domestic patents approved accounting for that applied. The reason why this paper uses the number of patent grants rather than that of applications is that there is a certain time lag effect in patent applications. And the meteorological (weather) conditions (‘wea’) are selected to represent the natural factors, which are represented by the proportion of the number of regional ecological and agricultural meteorological test stations accounting for the national totality.

#### Mechanism variables

The level of industrial development (‘ind’) is represented by the ratio of industrial-added value to regional GDP. Ecological construction and protection (‘eco’), the proportion of ecological construction and protection investment in regional GDP is selected to indicate that the improvement of ecological construction and protection investment can provide essential production factors for green development.

### Data sources and descriptive statistics

Based on the availability of data, the panel data of 30 provinces, municipalities, and autonomous regions in China (except Hong Kong, Macao, Taiwan, and Tibet) from 2013 to 2022 are selected as the sample. The required raw data are taken from 2014 to 2023 China Statistical Yearbook, China Rural Statistical Yearbook, China Agricultural Statistical Yearbook, the official authoritative data of provincial and municipal statistical bureaus, and the statistical data of China Green Food Development Center. The linear interpolation method is used to process the missing data. Table 2 gives the descriptive statistics of each variable.

**Table 2:** Descriptive statistics of main indicators.

Variables	Measure	Mean	Std. Dev.	Min	Max
AGD	AGD	0.203	0.080	0.053	0.434
*edu*	Education level	0.033	0.015	0.004	0.064
*inf*	Level of information development	0.033	0.028	0.003	0.168
*wea*	Meteorological conditions	0.033	0.015	0.001	0.069
*tec*	Science and technology level	0.033	0.046	0.000	0.232
*tecc*	Square term of the technological level	0.003	0.008	0.000	0.054
*ind*	Industrial development level	0.329	0.078	0.100	0.542
*eco*	Ecological protection efforts	0.004	0.003	0.000	0.014

Table 2 shows that the average value of AGD is 0.203, which means that the overall level of AGD is low, the minimum value is 0.053, the maximum value is 0.434, and there are some differences in the development of AGD among provinces. There are significant differences in the development of control variables and mechanism variables among regions.

## Empirical results

### Baseline regression results

In this paper, the regression is based on the benchmark model (Table 3). Column (1) is a DID regression without adding control variables; column (2) DID regression with non-fixed effects of control variables. The results show that ignoring the fixed effects will lead to large errors in the results. Column (3) is the DID regression that controls the time effect; column (4) is the DID regression of the two-way fixed effect, and it is also the benchmark regression result of this paper. The regression results of column (1)—(4) show that the DID coefficients of the core explanatory variables are positive and not much different, and the core explanatory variables in the model regression results are significant at the level of 1%, indicating that the green industrial policy has a significant positive impact on the AGD, that is, the “Implementation Opinions of the Ministry of Agriculture and Rural Affairs on Supporting the Green Development of Agriculture and Rural Areas in the Yangtze River Economic Belt” has a significant incentive effect on the Yangtze River Economic Belt. Hypothesis 1 is verified.

**Table 3:** The benchmark regression results of industrial policy on AGD.

Variables	AGD			
	(1)		(2)	
	Coefficient	SE	Coefficient	SE
did	0.025***	(0.006)	0.023***	(0.007)
*edu*			-2.566**	(1.222)
*inf*			1.056***	(0.360)
*wea*			2.025	(1.556)
*tec*			-0.724	(0.534)
*tecc*			2.826*	(1.660)
cons	0.157***	(0.005)	0.153**	(0.064)
Fixed time	Yes		Yes	
Fixed individual	Yes		Yes	
Control variable	No		Yes	
N	330	330	330	330

Note: The upper corner markers *, * * and * * * indicate significance at 10%, 5%, and 1% levels, respectively. The standard errors are in the brackets. (the same in the following tables).

The policy effect of the Yangtze River Economic Belt is 0.023. With the implementation of the “Implementation Opinions of the Ministry of Agriculture and Rural Affairs on Supporting the Green Development of Agriculture and Rural Areas in the Yangtze River Economic Belt”, the AGD of the Yangtze River Economic Belt has increased by 2.3 percentage points. The effect of education level on AGD is significant. Education can affect people’s cognitive level [73]. The higher the cognitive level of green life, the higher the willingness of people to adopt green production and lifestyle. However, at present, the main labor supply in rural areas is the left-behind elderly, which has a certain lag effect on the new concept and cognition of AGD. With the further improvement of education level, its positive impact will gradually become significant. The technical level has a significant effect on the right side of the positive U-shaped, and the square coefficients of the technical level and the technical level are − 0.724 and 2.826, respectively. In the early stage, the technical development has a certain crowding-out effect on AGD, but it is not significant. With the further improvement of the technical level, it plays a significant role in promoting AGD. The quality of meteorological conditions determines the yield of green agriculture to a certain extent. Accurate weather forecasts can provide important meteorological information for farmers to sow in time [74]. Column (4) shows that the positive coefficient of meteorological conditions is 2.205, but it is not significant. The possible explanation is that the establishment and development of ecological and agrometeorological experimental business stations started late. There were 67 nationwide in 2005, and there was little change in the next few years. It rose to 618 in 2012, remained stable in the next few years, and rose to 1195 in 2019 (data from the website of the National Bureau of Statistics). There is a certain time lag effect when it is put into operation. It is predicted that the improvement of ecological and agrometeorological experimental technology and the enhancement of technology transformation ability, will have a significant positive effect on AGD. The positive influence coefficient of the information development level is 1.056, which is significant at the level of 1%. The higher the level of information development, the more opportunities people have to contact new things [75], and the easier it is to form a new concept of green development, thus promoting the AGD. This also verifies the research of Zhao et al. [76], Luo et al. [77], Luh et al. [56] and Naseem et al. [78]. Zhao et al. (2023) found that the digital economy can promote green development by improving the education levels [76]. Luo et al. (2023) confirmed that scientific and technological innovation can promote the transformation of agriculture to green and low-carbon [77]. Luh et al. (2023) confirmed that climatic conditions play an important role in the development of organic agriculture [56]. Naseem et al. (2023) found that the improvement of information technology conditions can reduce carbon emissions in the agricultural sector [78].

### Robustness test

#### Parallel trend test

Using the DID to evaluate the implementation effect of the policy, only when the treatment group and the control group are sufficiently similar before the implementation of the policy, can the DID estimate be the causal effect of the policy, rather than the result of other factors. Therefore, it is necessary to carry out parallel trend hypothesis testing. There are two methods for parallel trend testing. The first is to draw the time trend chart of the mean of the explained variables of the treatment group and the control group, but this method is rough. To obtain more accurate and scientific conclusions, this paper uses the event research method [79] to construct the following regression Eq (2).


$${AGD}_{it}=\alpha +\sum_{\tau =1}^{6}\theta_{-\tau }D_{i,t-\tau }+\theta D_{it}+\sum_{\tau =1}^{4}\theta_{\tau }D_{i,t+\tau }+\rho X_{it}+\mu_{i}+\lambda_{t}+\varepsilon_{it}$$ (2)

**Fig 2:**
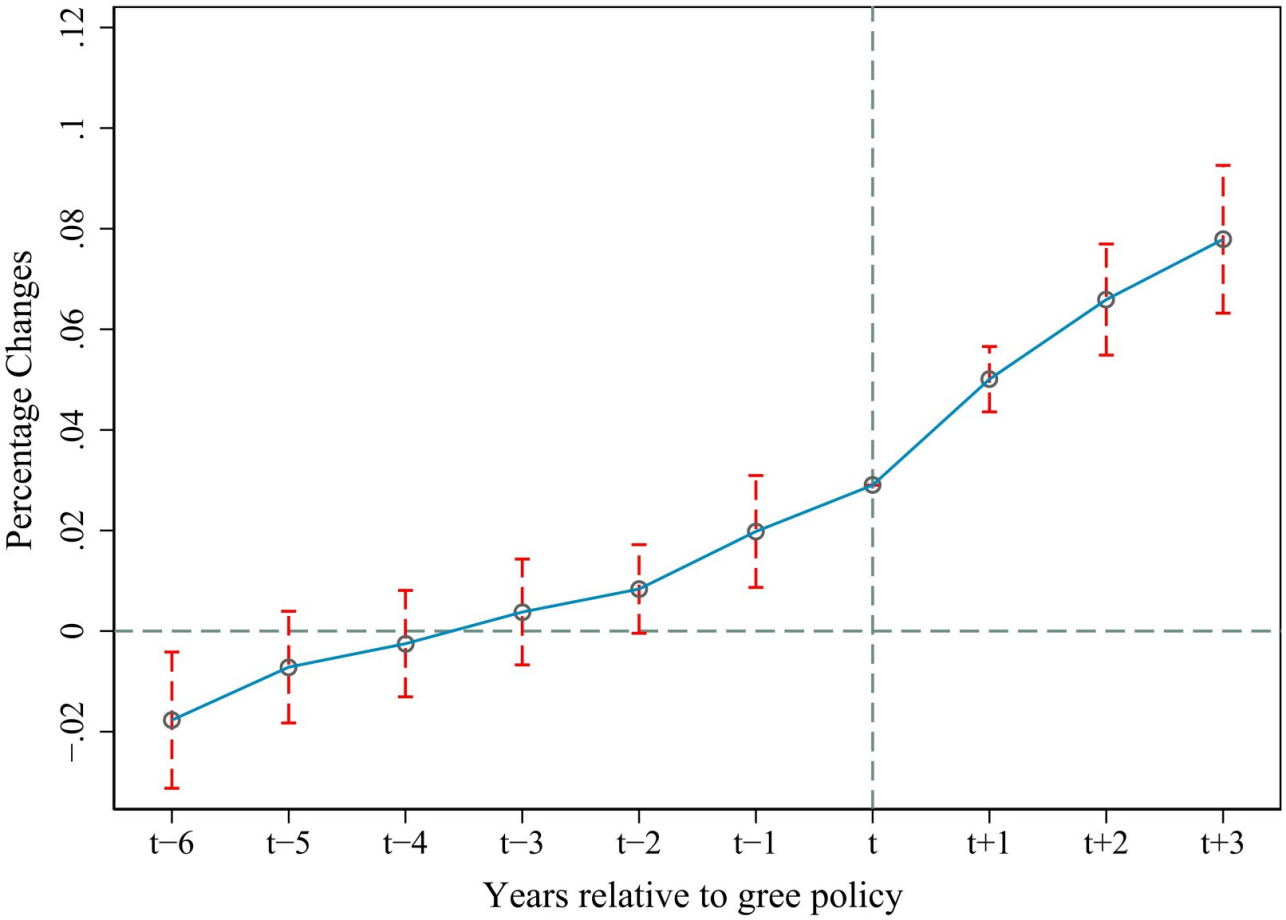
Parallel trend test. It shows that there is no significant difference between the treatment group and the control group before and after the implementation of the policy, which proves that the parallel trend hypothesis is satisfied. This figure was generated in STATA17.

In the model, there are 6 periods before the experiment and 4 periods after the experiment. θ represents the difference between before and after the experiment. This paper takes the year of policy formulation as the base period, and the lower corner τ represents the number of periods that differ from the current period of policy formulation. From Fig 2, it can be seen that before the implementation of the policy, the difference between the treatment group and the control group is almost not significant (the coefficient of the previous period of policy implementation is significant, and there may be a policy advance effect), that is, there is no systematic difference between the treatment group and the control group, which satisfies the parallel trend hypothesis. At the same time, it can also be seen that after the implementation of the “Implementation Opinions of the Ministry of Agriculture and Rural Affairs on Supporting the Green Development of Agriculture and Rural Areas in the Yangtze River Economic Belt”, the AGD difference between the treatment group and the control group became very significant, and the policy effect was getting better and better during the research period. It means that policy implementation has a significant incentive effect on AGD, and also verifies the robustness of the regression results.

#### Placebo test

**Fig 3:**
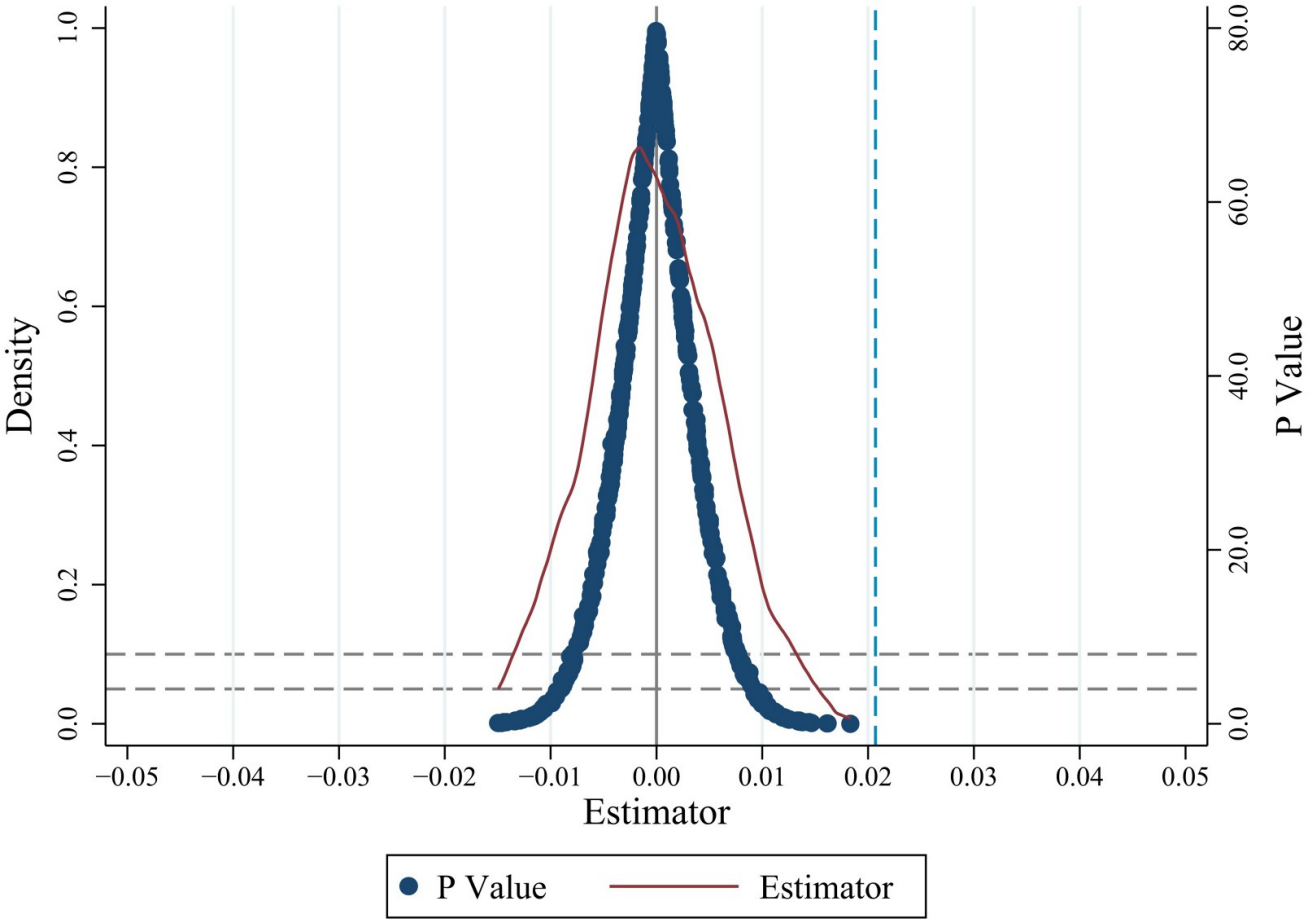
Randomly generated experimental group. The blue vertical dotted line on the right represents the real regression coefficient. It shows that the placebo test results are on the left side of the true value, and most of the estimated coefficients are not significant. It means that the original regression results are unlikely to be obtained by chance. This figure was generated in STATA17.

*(1) Randomly generated experimental group*. The placebo test focuses on the comparison with the true regression coefficient. In this paper, the placebo test is carried out by the method of random distribution. It can be seen from Fig 3 that the blue vertical dotted line on the right represents the real regression coefficient (that is, the regression based on the real nodes of the policy experiment). Most of the points of this placebo test fall on the left side of the real value. Most of the estimated p-values are greater than 0.05, and the coefficients are concentrated near 0. The distance between the mean and the real value is far, and most of the estimated coefficients are not significant, indicating that the effect of the shock is significant and not randomly generated, which means that the policy effect of the policy pilot area on AGD is not affected by other unobserved factors. It shows that our estimation results are unlikely to be obtained by chance, and therefore are unlikely to be affected by other policies or random factors.

*(2) The virtual control group is influenced by the policy*. To verify the accuracy of the results, this paper considers a place that is not affected by the policy and assumes that it is affected by the policy. It is a fictitious treatment group. If the regression result DID is significant, it is proved that the control group is also affected by the research policy, so the estimation results are biased. In Table 4, columns (1) and (2) are the regression results of the fictitious treatment group and the control variables added in turn. The results show that the core explanatory variable DID, that is, the policy effect, is not significant, thus verifying the robustness of the original benchmark regression results.

**Table 4:** Construct a placebo test of dummy variables.

Variables	AGD			
	(1)	(2)	(3)	(4)
did	-0.000 (0.009)	-0.002 (0.009)	0.011 (0.016)	0.011 (0.016)
cons	0.157*** (0.005)	0.158** (0.067)	0.157*** (0.005)	0.162*** (0.067)
Fixed time	Yes	Yes	Yes	Yes
Fixed individual	Yes	Yes	Yes	Yes
Control variable	No	Yes	No	Yes
N	330	330	330	330

*(3) Change the time when the policy occurs*. In this paper, through the fictitious policy occurrence time, that is, the policy occurrence time is assumed to be advanced to 2014, and the benchmark regression is carried out. If the policy time is advanced, the regression results of the estimator are still significant, then the original estimation results are likely to be biased. At this time, AGD is likely to be affected by other policy changes or random factors. Columns (3) and (4) in Table 4 assume that the time is advanced to 2014 and the regression results of the control variables are added in turn. The results show that the core explanatory variable DID, that is, the policy effect, is not significant, thus verifying the robustness of the original regression results.

*(4) Exclude the interference of other policies*. In the process of estimating the impact of the “Implementation Opinions of the Ministry of Agriculture and Rural Affairs on Supporting the Green Development of Agriculture and Rural Areas in the Yangtze River Economic Belt” on the AGD, it will inevitably be interfered with by other policies, so that the estimation results will be biased. To identify the possible interference of other policies, this paper searched the relevant policies on the AGD and found that in 2017, the General Office of the CPC Central Committee and the General Office of the State Council issued the “Opinions on Innovating Institutional Mechanisms to Promote AGD”, which is the first programmatic document issued by the Party Central Committee and the State Council to guide the AGD. In 2019, the Ministry of Agriculture and Rural Affairs of the People’s Republic of China formulated the “Work Points for Promoting the AGD and Rural Areas in the Yangtze River Economic Belt in 2019” to promote the AGD, so the estimated policy effect of this paper may be overestimated. To identify this impact, we assume that the experimental group is also affected by this policy, and add the policy dummy variables of 2017 and 2019 respectively. If the regression results are not significant, it shows that the conclusions of this paper are biased. If the regression results are significant but the DID coefficient is reduced, it shows that the policy effect presented by the original regression results is overestimated, which proves that it is also affected by other policies, but it shows that the original regression results of this paper are relatively robust [80]. In Table 5, columns (1) and (2) are the regression results of policy impact in 2017 and adding control variables in turn. Columns (3) and (4) are the regression results of policy impact in 2019 and adding control variables in turn. The regression results show that the regression results of adding policy dummy variables in 2017 and 2019 are significant, but the coefficients are lower than the original benchmark regression coefficient of 0.023. Therefore, the policy effect is indeed overestimated, but the incentive effect of the research policy on AGD still exists, which verifies the robustness of the regression results in this paper.

**Table 5:** Exclude other policy interference regression results.

Variables	AGD			
	(1)	(2)	(3)	(4)
did	0.019** (0.007)	0.016* (0.008)	0.019*** (0.006)	0.018*** (0.007)
cons	0.157*** (0.004)	0.132** (0.066)	0.157*** (0.005)	0.140** (0.065)
Fixed time	Yes	Yes	Yes	Yes
Fixed individual	Yes	Yes	Yes	Yes
Control variable	No	Yes	No	Yes
N	330	330	330	330

### Mechanism analysis

The above analysis shows that the green industrial policy can effectively promote the AGD of the Yangtze River Economic Belt. To verify Hypothesis 2 and Hypothesis 3, Based on the idea of Wang et al. [81], a model is constructed, and a two-step regression method is used to test whether the green industrial policy has a crowding-out effect on industrial development and whether it improves ecological construction and protection, thus promoting AGD.


$${AGD}_{it}=\alpha +\beta {DID}_{it}+\rho X_{it}+\mu_{i}+\lambda_{t}+\varepsilon_{it}$$ (3)

$${ind}_{it}=\alpha_{1}+\beta_{1}{DID}_{it}+\rho_{1}X_{it}+\mu_{i}+\lambda_{t}+\varepsilon_{it1}$$ (4)

$${eco}_{it}=\alpha_{2}+\beta_{1}{DID}_{it}+\rho_{2}X_{it}+\mu_{i}+\lambda_{t}+\varepsilon_{it2}$$ (5)

Among them, Eq (3) is the benchmark regression, Eqs (4) and (5) are the mechanism regression. If both *β*_1_ and *β*_2_ are significant, it shows that the research policy in this paper significantly affects the level of industrial development and ecological construction and protection, hypotheses 2 and 3 are proved. Table 6 shows the regression results of the mechanism. Overall, the policy effect is significant regardless of whether the control variables are added. Column (1) is the benchmark DID regression, and columns (2) and (3) are the regression results for the mechanism variable *ind*, indicating that the “Implementation Opinions of the Ministry of Agriculture and Rural Affairs on Supporting the Green Development of Agriculture and Rural Areas in the Yangtze River Economic Belt” has significantly weakened industrial development, with a negative effect of 0.023. The implementation of green industrial policy has a certain crowding-out effect on industrial development, which makes green agriculture get more policy tilt and attention, to promote the AGD. Columns (3) and (4) are the regression results of the mechanism variable *eco*, indicating that the “Implementation Opinions of the Ministry of Agriculture and Rural Affairs on Supporting the Green Development of Agriculture and Rural Areas in the Yangtze River Economic Belt” has significantly promoted the level of ecological protection and investment, and the promotion effect is 0.001. The increase in the proportion of investment in ecological construction and protection can provide corresponding production factors such as capital for the AGD [82], which is conducive to promoting the AGD. Therefore, the hypothesis is proved.

**Table 6:** Mechanism test regression results.

Variables	AGD	*ind*		*eco*	
	(1)	(2)	(3)	(4)	(5)
did	0.023*** (0.007)	-0.019*** (0.0057)	-0.023*** (0.005)	0.001*** (0.000)	0.001*** (0.000)
cons	0.153** (0.064)	0.384*** (0.004)	0.169*** (0.053)	0.004*** (0.000)	0.009*** (0.002)
Fixed time	Yes	Yes	Yes	Yes	Yes
Fixed individual	Yes	Yes	Yes	Yes	Yes
Control variable	Yes	No	Yes	No	Yes
N	330	330	330	330	330

## Heterogeneity discussion

It can be seen from Table 7 that the policy effect of the upper reaches of the Yangtze River Economic Belt is 0.041, which is significant at the level of 1%, and the policy effect of the middle reaches of the Yangtze River Economic Belt is not significant. The policy effect of the lower reaches of the Yangtze River Economic Belt is 0.044, which is significant at the level of 5%. It shows that the policy has a significant positive incentive effect on the AGD in the upstream and downstream of the Yangtze River Economic Belt. The upper reaches of the Yangtze River Economic Belt include Chongqing, Sichuan, Guizhou, and Yunnan. Although the economic foundation of these upstream areas is weak, they have good resource endowments and rich ecological resources such as “green water and green mountains” [45]. There is a large space for the development of “green water and green mountains” into “golden mountains and silver mountains”, so the policy incentive effect is very significant. The lower reaches of the Yangtze River Economic Belt include Shanghai, Jiangsu, and Zhejiang. The environmentally friendly index of the economic development process in the downstream region is low [30], and the agricultural non-point source pollution caused by it also challenges the carrying capacity of resources and the environment. Economic development is constrained by resources and the environment. Excessive use of agricultural inputs such as fertilizers and pesticides, unreasonable disposal of agricultural wastes such as livestock and poultry manure, crop straw, and farmland residual film [38]. In particular, it is difficult to control agricultural non-point source pollution in Zhejiang. However, these downstream areas have a good economic foundation, and the ability of scientific and technological innovation is outstanding. The optimization of industrial structure and urbanization rate is better than those in the upstream and middle reaches [83]. The ability of scientific and technological transformation is strong, and the relationship between the development of the technical level and AGD is on the right side of the positive U-shaped, that is, there are considerable technical conditions to control agricultural pollution and improve the level of green development. The middle reaches of the Yangtze River Economic Belt include Anhui, Jiangxi, Hubei, and Hunan. The economic development level of these middle reaches is between the upper and lower reaches. The industrial pillar is mainly the secondary industry, and the ability of scientific and technological innovation is general [27]. Industrial pollution puts greater pressure on resources and the environment. In other words, the middle reaches have dual pressures of economy and environment and lack prominent advantages. Industrial pollution has a greater negative effect on the AGD, and the effect of policy incentives is not obvious in a short period. This also verifies the research of Ding et al. [84], Yuan et al. [85] and Wang et al. [83]. Ding et al. (2021) confirmed that the overall utilization efficiency of agriculture in the Yangtze River Economic Belt is on the rise, but it is better in the eastern cities and worse in the central cities [84]. Yuan et al. (2022) confirmed that there is a large gap in green development between regions within the Yangtze River Economic Belt [85]. Wang et al. (2023) confirmed that the appropriate formulation of land ecological protection policies can promote the coordinated development of the upper, middle, and lower reaches of the Yangtze River [83].

**Table 7:** Heterogeneous regression results.

Variables	Upper reaches of the Yangtze River		Middle reaches of the Yangtze River		Lower reaches of the Yangtze River	
	(1)	(2)	(3)	(4)	(5)	(6)
did	0.034*** (0.009)	0.041*** (0.010)	0.023 (0.017)	0.012 (0.017)	0.057*** (0.017)	0.044** (0.017)
*edu*		-4.501*** (1.317)		-2.159* (1.272)		-1.496 (1.278)
*inf*		1.144*** (0.352)		1.276*** (0.365)		1.096*** (0.367)
*wea*		1.971 (1.541)		1.560 (1.587)		1.600 (1.568)
*tec*		-0.443 (0.538)		-0.887 (0.543)		-0.679 (0.543)
*tecc*		1.563 (1.675)		2.854* (1.695)		2.174 (1.698)
cons	0.157*** (0.005)	0.212*** (0.065)	0.157*** (0.005)	0.153** (0.066)	0.157*** (0.005)	0.131** (0.066)
Fixed time	Yes	Yes	Yes	Yes	Yes	Yes
Fixed individual	Yes	Yes	Yes	Yes	Yes	Yes
Control variable	No	Yes	No	Yes	No	Yes
N	330	330	330	330		

## Conclusions and policy suggestions

### Conclusions

Based on the panel data of 30 provinces, municipalities, and autonomous regions in China from 2012 to 2022, from the perspective of harmony between man and nature, this paper selects 20 indicators to measure the level of AGD from five dimensions such as ecological conservation and resource conservation by entropy weight method. On this basis, taking the implementation of green industrial policy in the Yangtze River Economic Belt as a quasi-natural experiment, the policy effect of green industrial policy on AGD was analyzed by using the DID method.

The research shows that: (1) the AGD is increasing year by year in each province, but there are some differences in the AGD among provinces; (2) Compared with the non-implementation areas of policies, the AGD in the implementation areas of policies has been significantly improved, and has passed a series of robustness tests; (3) The mechanism analysis shows that the green industrial policy has a crowding-out effect on industrial development, but significantly enhances the ecological construction and protection, thus promoting the AGD; (4) Heterogeneity analysis shows that the policy has a significant positive incentive effect on the upper and lower reaches of the Yangtze River Economic Belt, and the incentive effect on the middle reaches is not significant; (5) The impact of technological level on AGD shows a positive U-shaped relationship. The improvement of education and information development levels also effectively promotes the AGD. AGD is not achieved overnight. It requires people to have a series of chain reactions from cognition-concept-agricultural green consumption mode-agricultural green production behavior. Therefore, AGD is not easy under the new situation. Based on the existing research, this paper deepens the understanding of AGD by policy. The research conclusion contains important policy significance, provides a valuable reference for the formulation and optimization of green industrial policy in the future, and has important practical significance for promoting the green and low-carbon development of agriculture.

### Policy suggestions

Under the guidance of the new development concept, choosing a suitable green industrial policy will provide a new path and direction for China’s AGD and industrial structure optimization and upgrading. Based on the theoretical and empirical analysis of the article, as well as the process of agricultural power and greening, the following policy recommendations are proposed.

Firstly, balance the differences in AGD among provinces. They should strengthen the cooperation and exchange of AGD among provinces, promote the dissemination of AGD experience of advanced provinces to other provinces, encourage cross-regional cooperation in AGD projects, and promote resource sharing and complementary advantages. At the same time, according to the resource endowment and agricultural characteristics of different provinces, differentiated AGD planning and support policies are formulated. In addition, they should increase investment in agricultural green technology innovation, improve the technical level of AGD, and reduce regional differences with scientific and technological support.

Secondly, strengthen policy implementation and evaluation. China should introduce more accurate regional AGD strategies and policies, and formulate differentiated support policies according to the natural, economic and social conditions of different regions. For areas with low levels of AGD, increase financial transfer payments, provide technical, talent and financial support, and help them improve the level of green development. Ensure the effective implementation of green agriculture policies in various places, and strengthen policy implementation and supervision mechanisms. At the same time, the effect of policy implementation is evaluated regularly, and the policy content is adjusted and improved in time according to the evaluation results. Through policy publicity and training, farmers’ awareness and participation in green agricultural policies are improved.

Thirdly, optimize the industrial structure and promote the AGD. While ensuring industrial development, they should pay attention to the formulation and implementation of green industrial policies, reduce the crowding-out effect on industrial development, strengthen the integration and development of agriculture, industry, service industry and other industries, and promote the extension and expansion of agricultural industrial chain. In addition, ecological construction and protection should be strengthened to improve the stability and sustainability of agricultural ecosystems. Moreover, they should encourage and support the research and development and application of agricultural green production technology, guide and promote the development of emerging industries and high-tech industries, especially in areas related to AGD, such as ecological agriculture, circular agriculture, and precision agriculture, so as to improve the green level of agricultural production.

Fourthly, formulate differentiated policies for different regions of the Yangtze River Economic Belt. For the upper and lower reaches of the Yangtze River Economic Belt, the government should continue to increase policy support to consolidate and expand the achievements of AGD. For the middle reaches, the government should formulate targeted AGD policies, such as providing preferential policies such as finance and taxation, and encouraging farmers and enterprises to participate in green agricultural production. At the same time, they should increase investment in AGD in the middle reaches, support the research and development and promotion of green agricultural technology, and improve agricultural production efficiency. In addition, the demonstration area of AGD can be built in the middle reaches. Through the construction of the demonstration area, the development achievements and advantages of green agriculture can be displayed, and the achievements of AGD can be evaluated and summarized regularly, and the problems can be found and the policies can be improved and adjusted in time.

Fifthly, improve the level of technology, education and information development. Relevant subjects should increase investment in R&D of agricultural green technology, improve the level of technology, and promote the innovation and application of agricultural green technology. At the same time, they should strengthen agricultural education and training, improve the cultural quality and scientific and technological level of farmers, guide farmers to establish the concept of green production, consciously practice green production behavior, cultivate modern farmers with the concept of green production, and form a good atmosphere for the whole society to participate in the AGD. In addition, China should strengthen the construction of rural informatization, improve the speed and coverage of information dissemination, and promote the sharing and utilization of agricultural information.

In short, under the new situation, people should fully realize the importance of AGD. Given the differences and spatial correlation of AGD in different regions, the state should provide differentiated policy support and financial assistance for AGD. According to the actual situation of local agricultural development and resources, each region should strengthen the concept of green development, take the green transformation of agriculture as the core of current agricultural work, actively explore the specific ways of AGD, summarize the relevant experience, continuously optimize the institutional mechanism of the AGD, and improve macro planning. At the same time, it is necessary to solve the problems at the level of system and mechanism, and increase investment in green agricultural science and technology innovation, to provide impetus for green sustainable development of agriculture by innovation-driven.

### Limitations and future research

Based on the availability of data, this paper only explores the policy effect of AGD from the national macro level, and the index system of AGD level is not perfect. Therefore, it will be the next research direction to find suitable data substitution for difficult-to-obtain data to carry out micro-analysis and improve its index system, and on this basis to further explore the AGD in specific counties or towns.

## Supporting information

S1 Data (ZIP)
